# Imaging analysis of human metapneumovirus-infected cells provides evidence for the involvement of F-actin and the raft-lipid microdomains in virus morphogenesis

**DOI:** 10.1186/s12985-014-0198-8

**Published:** 2014-11-19

**Authors:** Muhammad Raihan Jumat, Tra Nguyen Huong, Puisan Wong, Liat Hui Loo, Boon Huan Tan, Fiona Fenwick, Geoffrey L Toms, Richard J Sugrue

**Affiliations:** Division of Molecular Genetics and Cell Biology, School of Biological Sciences, Nanyang Technological University, 60 Nanyang Drive, Nanyang, 637551 Republic of Singapore; Detection and Diagnostics Laboratory, DSO National Laboratories, 27 Medical Drive, Singapore, 117510 Republic of Singapore; School of Clinical Medical Sciences, The University of Newcastle, Newcastle upon Tyne, NE24HH UK

**Keywords:** Human metapneumovirus, Paramyxovirus, Virus filaments, Virus assembly, Lipid-raft, F-actin

## Abstract

**Backgound:**

Due to difficulties of culturing Human metapneumovirus (HMPV) much of the current understanding of HMPV replication can be inferred from other closely related viruses. The slow rates of virus replication prevent many biochemical analyses of HMPV particles. In this study imaging was used to examine the process of HMPV morphogenesis in individually infected LLC-MK2 cells, and to better characterise the sites of HMPV assembly. This strategy has circumvented the problems associated with slow replication rates and allowed us to characterise both the HMPV particles and the sites of HMPV morphogenesis.

**Methods:**

HMPV-infected LLC-MK2 cells were stained with antibodies that recognised the HMPV fusion protein (F protein), attachment protein (G protein) and matrix protein (M protein), and fluorescent probes that detect GM1 within lipid-raft membranes (CTX-B-AF488) and F-actin (Phalloidin-FITC). The stained cells were examined by confocal microscopy, which allowed imaging of F-actin, GM1 and virus particles in HMPV-infected cells. Cells co-expressing recombinant HMPV G and F proteins formed virus-like particles and were co-stained with antibodies that recognise the recombinant G and F proteins and phalloidin-FITC and CTX-B-AF594, and the distribution of the G and F proteins, GM1 and F-actin determined.

**Results:**

HMPV-infected cells stained with anti-F, anti-G or anti-M revealed a filamentous staining pattern, indicating that the HMPV particles have a filamentous morphology. Staining of HMPV-infected cells with anti-G and either phalloidin-FITC or CTX-B-AF488 exhibited extensive co-localisation of these cellular probes within the HMPV filaments. This suggested that lipid-raft membrane domains and F-actin structures are present at the site of the virus morphogenesis, and are subsequently incorporated into the HMPV filaments. Furthermore, the filamentous virus-like particles that form in cells expressing the G protein formed in cellular structures containing GM1 and F-actin, suggesting the G protein contains intrinsic targeting signals to the sites of virus assembly.

**Conclusions:**

These data suggest that HMPV matures as filamentous particles and that virus morphogenesis occurs within lipid-raft microdomains containing localized concentrations of F-actin. The similarity between HMPV morphogenesis and the closely related human respiratory syncytial virus suggests that involvement of F-actin and lipid-raft microdomains in virus morphogenesis may be a common feature of the *Pneumovirinae*.

**Electronic supplementary material:**

The online version of this article (doi:10.1186/s12985-014-0198-8) contains supplementary material, which is available to authorized users.

## Background

Human metapneumovirus (HMPV) is a new member of the *Paramyxoviridae* that was first identified in children with respiratory diseases in Netherlands [1]. The clinical symptoms that are caused by HMPV infections in children are similar to those observed with respiratory syncytial virus (RSV) infection, ranging from upper respiratory tract infection to bronchiolitis and pneumonia. HMPV has become recognised as a major cause of lower respiratory infection in children [2,3].

The mature HMPV particle is surrounded by a lipid envelope in which the virus fusion (F) and attachment (G) proteins are inserted. The F protein mediates fusion of the virus and host cell membranes during virus entry [4], while a primary role for the G protein in virus attachment to susceptible cells has been demonstrated [5]. The virus envelope surrounds a protein layer formed by the matrix (M) protein, and a ribonucleoprotein (RNP) complex that is formed by the viral genomic RNA (vRNA), the nucleocapsid (N) protein, the phosphoprotein (P protein), the M2-1 protein and the large (L) protein [6]. Based on genetic analysis of HMPV genome sequences two major HMPV genotypes, called HMPV A and B, have been identified [7–9].

Much of the current understanding of the biology of the HMPV can be inferred from other closely related viruses e.g. RSV and avian pneumovirus [7,10]. Primary isolation of HMPV has been achieved in several different cell lines [8,11,12], and some tissue culture adapted isolates have been described [8]. However, their cultivation can require up to 14 days incubation before cytopathic effects are visualised [12]. This low level of virus replication in standard cell culture, particularly low-passaged clinical isolates, and the subsequent recovery of low levels of infectious HMPV, have hampered functional biochemical studies on the virus. These studies usually require higher levels of biological material that can be achieved following a single cycle of HMPV replication.

Visualising the distribution of individual virus structural protein is a prerequisite for understanding the process of HMPV maturation, and *in situ* imaging of virus-infected cells stained using virus protein specific antibodies is in general the most direct and unambiguous method to do this. Therefore, in this current study we have circumvented the problems associated with low virus replication rates by using imaging to examine HMPV morphogenesis. This has allowed us to visualize the morphogenesis of a low passaged HMPV clinical isolate in mammalian tissue culture, and to suggest a role for lipid-raft microdomains and F-actin in the process of HMPV maturation.

## Results and discussion

### HMPV assembles as filamentous structures on virus-infected LLC-MK2 cells

The HMPV A2 strain NCL03-4/174 used in this study was isolated from nasal secretions of children with respiratory infection, and the virus was cultured as described previously [13]. HMPV isolation and propagation in the LLC-MK2 cell line has been described by several groups [12,14,15], and this cell line was used throughout our study. The distribution of several major virus structural proteins was characterised using antibodies against the F, G, and M proteins to stain HMPV-infected cells. The preparation of the HMPV F (MAb34 and MAb58) and G (MAbAT1) protein antibodies has been described previously [16], and the antibody against the HMPV M protein (anti-M) was prepared using bacterially expressed recombinant HMPV M protein.

Semi-confluent cell monolayers were infected with HMPV using a multiplicity of infection (moi) of 0.05, and at 3, 7 and 10 days-post infection (dpi) the monolayers were fixed and stained using anti-M (Additional file 1: Figure S1). By 3 dpi we were able to detect individually infected cells which exhibited low levels of anti-M staining. However, by 7 dpi infected cells showed increased levels of fluorescence staining, and the appearance of brightly stained infected cell clusters within the monolayer was noted by 10 dpi. Interestingly, the low rates of infection of the HMPV NCL03-4/174 isolate in the LLC-MK2 cell monolayer suggested that although the virus can infect these cells it is not extensively tissue culture-adapted. At 14 dpi approximately 1.8x10^2^ infectious virus particles per ml was detected in the tissue culture supernatant of HMPV-infected cells. However, the infected cell clusters were the most prominent staining pattern detected on the cell monolayers, and the increase in cluster size was time-dependant. This provided evidence that HMPV transmission occurred by localised transmission within the LLC-MK2 cell monolayer, in a manner similar to that described in RSV-infected cell monolayers [17].

**Figure 1:**
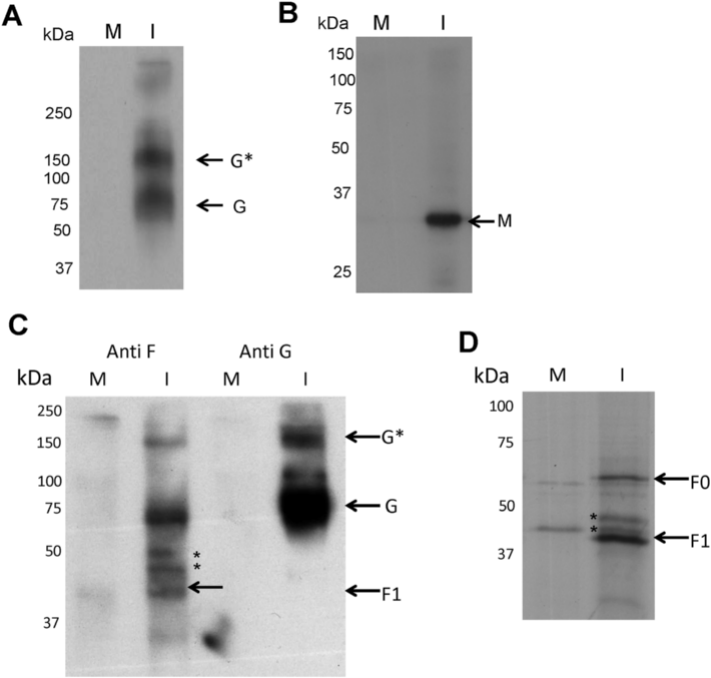
**Analysis of G, F and M proteins in HMPV-infected cells.** At 10 days post-infection (dpi) mock-infected (M) and HMPV-infected (I) cells were extracted in boiling mix and analysed by immunoblotting with **(A)** anti-G or **(B)** anti-M. Protein species corresponding in size to G protein (G) and a larger G protein species (G*), and the matrix protein (M) are indicated. **(C and D)**. At 10 dpi mock-infected (M) and HMPV-infected (I) cells were surface-biotinylated using sulpho-NHS-LC-LC-biotin as described previously ( [18]). The surface-labelled detergent extract was immunoprecipitated using **(C)** MAbAT1 (anti-G) or MAb58 (anti-F). The biotin-labelled proteins were immunoblotted and detected using strepavidin-HRP. Surface-biotinylated protein species corresponding in size to G, G*, and the F1 subunit (F1) are indicated. **(D)** Mock and HMPV-infected cells at 10 dpi were labelled for 16 hr using 100 μCi/ml L-[35 S]-methionine (EasyTag, PerkinElmer) in methionine free-DMEM (Invitrogen) and the detergent extracts immunoprecipitated using MAb58 as described previously [34]. The F0 and F1 subunit are indicated. In plate C and D additional protein products that are immunoprecipitated with anti-F are indicated (*).

Since 10 dpi allowed clear visualisation of infected cells by antibody staining without extensive CPE, all our subsequent analyses were performed at this time of infection unless otherwise stated. To demonstrate the specificity of the antibodies used in this study, detergent extracts were prepared from mock-infected and virus-infected cells, and the virus proteins examined by immunoblotting and immunoprecipitation (Figure 1). Mock and HMPV-infected cells were detergent exacted and the presence of the G protein detected by immunoblotting using MAbAT1. This revealed the presence of the 70 kDa monomeric (G) and a 170 kDa G protein species (G*) (Figure 1A). It is currently unclear if G* is a differentially glycosylated form of the G protein or if it is an oligomeric form of the G protein that shows increased resistance to heat denaturation. Immunoblotting of detergent extracts prepared from mock and HMPV-infected cells with anti-M revealed the presence of a 30 kDa protein in the infected cells, consistent with the presence of the M protein (Figure 1B). In addition the cells were surface-biotinylated, detergent-extracted, and the surface-labelled G and F proteins isolated from the detergent extract by immunoprecipitation using MAbAT1 and MAb58 respectively as described previously [18]. Immunoprecipitation with MAbAT1 revealed the presence of G and low levels of G* (Figure 1C), while immunoprecipitation with MAb58 revealed the presence of the 45 kDa F1 subunit similar to that described previously [4]. Immunoprecipitation with MAb58 also revealed the presence of an additional 70 and a 170 kDa surface biotinylated species that appeared to co-precipitate with the F1 subunit. The F0 precursor and F1 protein subunit were also detected in [S^35^] methionine-labelled HMPV-infected cells immunoprecipitated with MAb58 (Figure 1D).

**Figure 2:**
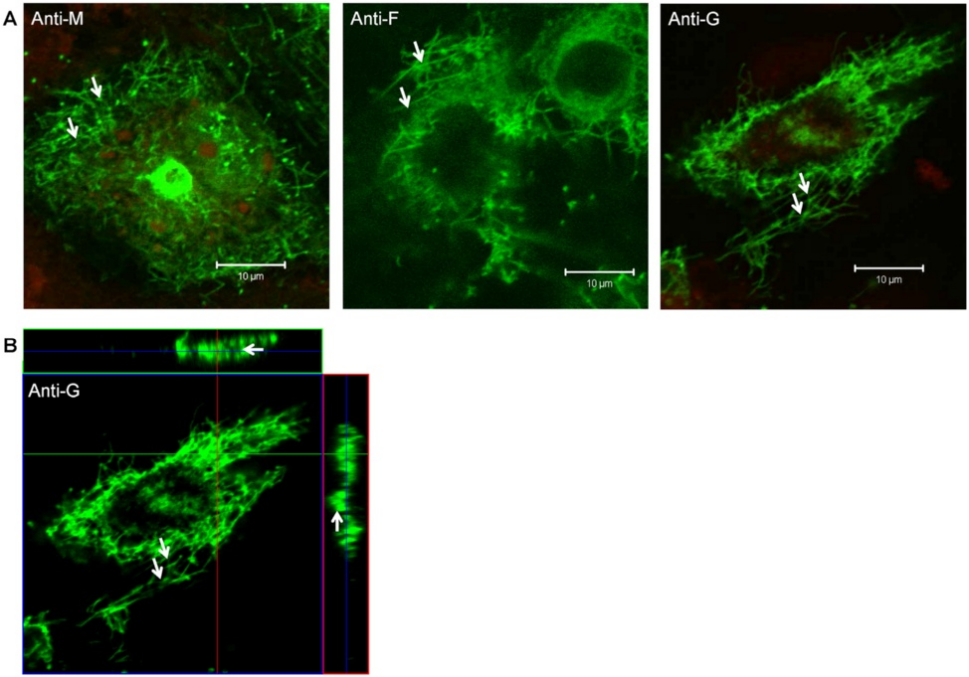
**Virus filament formation occurs on human metapneumovirus (HMPV)-infected LLC-MK2 cells.** Confocal microscopy imaging of HMPV-infected LLC-MK2 cells (at 10 days post-infection) probed with **(A)** anti-M, anti-F and anti-G (green). The cells were also co-stained using Evans Blue (red). **(B)** Cross-section image of anti-G stained HMPV-infected LLC-MK2 cells showing virus filaments. In each case the virus filaments are indicated (white arrows).

HMPV-infected cells stained using anti-M, anti-F or anti-G were examined using confocal microscopy at an optical plane that allowed imaging of the cell surface. Staining with each of the three different antibodies revealed a similar filamentous staining pattern in each case (Figure 2A). This indicated that HMPV particles can form filamentous structures in a manner similar to that described for the mature RSV particles on infected cells [19–21]. Examination of anti-G stained cells in cross-section further confirmed the presence of these filamentous structures on the surface of infected cells (Figure 2B). In a parallel analysis, infected cells were stained using anti-G and Evans Blue (Additional file 1: Figure S1B), the latter being a non-specific counter stain that is used to stain the cell monolayer (i.e. both non-infected and infected). It was noted that these filamentous virus structures appeared to spread from the infected cells to the surrounding non-infected cells. Although the significance of this vis-a-vis HMPV transmission is unclear, in the closely related RSV, virus filaments have been shown to play a direct role in virus cell-to-cell transmission [17].

**Figure 3:**
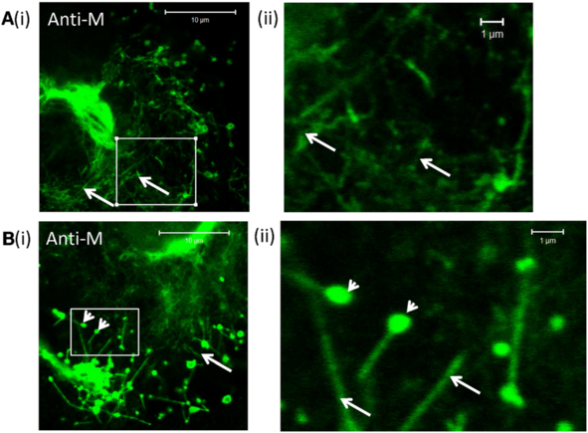
**HMPV-infected cells exhibit two specific sub-populations of the M protein.** Human metapneumovirus (HMPV)-infected LLC-MK2 cells were stained using anti-M, and imaged by confocal microscopy. **(A)** The virus filaments (white arrows) and **(B)** spherical M protein staining pattern (arrow heads) are highlighted. **(ii)** is an enlarged image taken from the area highlighted by the white box in **(i)**.

Interestingly, closer inspection of the anti-M stained HMPV-infected cells revealed that in addition to a filamentous staining pattern (Figure 3A), the presence of a spherical staining pattern at the ends of some of these filaments (Figure 3B). These varied in size, but we estimated they had an approx. diameter of between 400 and 800 nm, and were located most commonly around the periphery of the infected cells (Figure 3B(ii), highlighted by white arrow heads). Infected cells stained using anti-F or anti-G did not show this alternative prominent staining pattern. A comparison of the surface topology of mock-infected and HMPV-infected cells was performed using scanning electron microscopy (SEM) (Additional file 2: Figure S2). This confirmed the presence of HMPV-induced filamentous structures on the surface of infected cells; and was consistent with filamentous staining pattern observed using light microscopy. Interestingly, the SEM analysis also showed the presence of spherical structures associated with the virus filaments, and which may represent the locations of the M protein special structures detected by confocal microscopy.

The significance of the spherical M protein staining pattern is unclear, but does suggest that in addition to the virus particle associated M protein, a second population of the M protein that does not show a similar staining to that observed using either anti-G or anti-F staining. This alternative M protein staining pattern may be related to the cell-free form of the M protein that has been described in HMPV-infected cells [22]. In addition, our previous studies employing recombinant expression to generate HMPV virus like particles (VLPs) [23] identified a population of the HMPV M protein that was secreted within membrane vesicles but which did not co-purify with the VLPs [23].

### The sites of HMPV morphogenesis contain F-actin and GM1

The role of lipid-raft membranes and the cortical F-actin network during RSV morphogenesis has been described [24–28]. We therefore examined if F-actin and lipid-raft microdomains were also associated with HMPV filaments during virus assembly. Phalloidin-FITC binds to F-actin and cholera toxin-B subunit (CTX-B-AF488) binds to the raft-lipid GM1, enabling the detection of F-actin and lipid-raft membranes respectively. These fluorescence probes were used to examine the distribution of F-actin and lipid-raft membranes with respect to the HMPV filaments in HMPV-infected cells.

**Figure 4:**
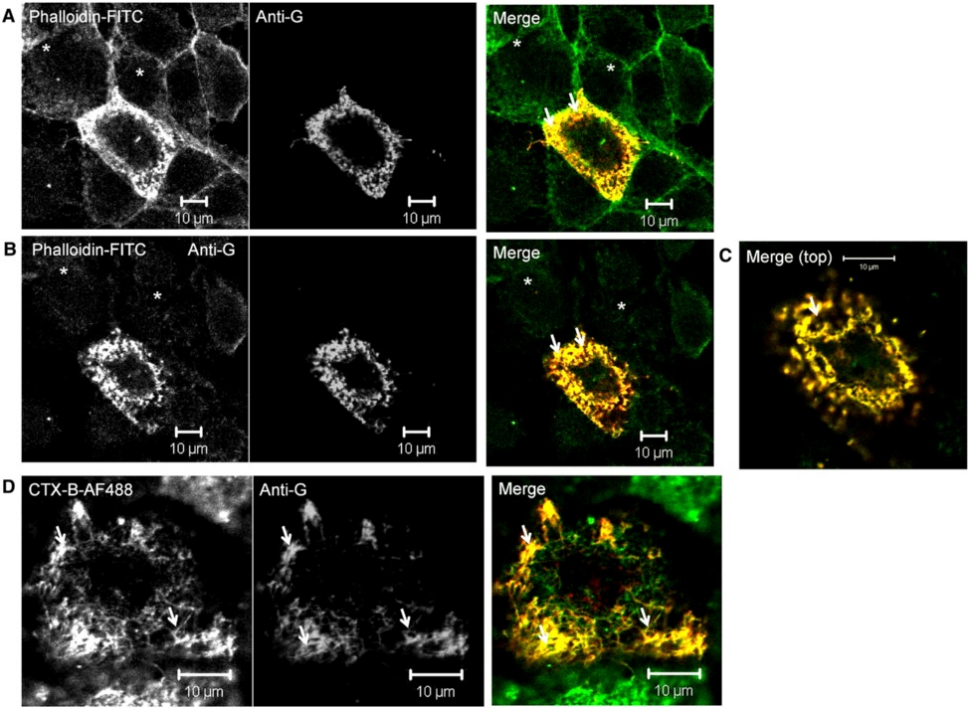
**Human metapneumovirus (HMPV) morphogenesis occurs within membrane structures enriched in lipid-rafts and F-actin.** At 7 days post-infection HMPV-infected LLC-MK2 cells were co-stained with phalloidin-FITC and anti-G and imaged using confocal microscopy at a focal plane that allowed visualisation of the **(A)** cell periphery and **(B)** cell top. **(C)** is an enlarged image of the cell viewed in **(B)**. **(D)** HMPV-infected LLC-MK2 cells were stained with CTX-B-AF488 and anti-G and imaged using confocal microscopy. In each case yellow staining pattern in the merged image indicates regions of co-localisation. The virus filaments (white arrows) and non-infected cells (*) are highlighted.

Cells were infected with HMPV using a moi of 0.05 and at 7 dpi the cells were stained using phalloidin-FITC and anti-G. The stained cells were imaged using confocal microscopy at optical planes which allowed the cell periphery (Figure 4A) and cell top (Figure 4B and C) to be visualized. The stained monolayer allowed the detection of both infected and non-infected cells, and we noted a similar prominent filamentous staining pattern for both anti-G and phalloidin-FITC in HMPV-infected cells. The LSM510 software was used to measure the degree of co-localisation within this filamentous staining pattern. A Mander’s coefficient of 1.0 ± 0.0 and Pearson’s coefficients of 0.82 ± 0.05 indicated high levels of co-localisation in these filamentous structures. Our imaging analysis suggested that F-actin was present within the HMPV filaments in a similar manner to that described for RSV [17,28,29].

Due to the low replication rates of HMPV not all cells in the monolayer were infected at 7 dpi. This enabled us to distinguish the infected cells (anti-G stained positive) from the remaining non-infected cells (anti-G stained negative) within the same cell monolayer (Figure 4A). It was noted that this prominent filamentous F-actin staining pattern was not detected in non-infected cells at a similar optical plane, suggesting that HMPV infection may alter the structure of the cortical actin network in a similar manner to that described in RSV-infected cells [25,28,30]. We also observed these co-stained filamentous structures spreading to the non-infected cells, suggesting that F-actin may play a role in virus transmission in the monolayer, in a similar manner to that proposed for RSV [17,25,30].

HMPV-infected cells were stained with anti-G and CTX-B-AF488, and examined using confocal microscopy at an optical plane that allowed the virus filaments to be visualized (Figure 4D). We observed a similar filamentous staining pattern for both CTX-B-AF488 and anti-G, and a high level of co-localisation within this filamentous staining pattern as indicated by both Mander’s coefficient and Pearson’s coefficients of 1.00 ± 0.0 and 0.83 ± 0.04 respectively. Our data also suggested that HMPV assembly occurs within membrane microdomains that are enriched in lipid-rafts, and that these lipids are incorporated into the virus envelope.

### The HMPV G protein preferentially localises to cellular microdomains enriched in F-actin and GM1

Using recombinant expression we have recently demonstrated that co-expression of the HMPV G and F proteins in LLC-MK2 cells leads to the formation of virus-like particles (VLPs) [23]. These VLPs form filamentous structures on the surface of transfected cells that are smaller but similar in appearance to the filamentous HMPV particles. We noted that expression of the G protein was sufficient to form VLPs, and VLPs formed in cells co-expressing the G and F proteins contained a protein complex involving the HMPV F and G proteins. We interpreted these VLP structures as indicating sites of HMPV assembly to which the G protein and F proteins were trafficked, rather than representing the formation of mature virus particles. This further suggested that the G and F proteins contain trafficking signals that allow their targeting to these sites of virus assembly. By extrapolation we rationalised that these signals would also play a role in their incorporation into HMPV particles when they form in HMPV-infected cells. On this basis we concluded that the recombinant G protein could be used as a marker with which to identify the sites of virus assembly.

**Figure 5:**
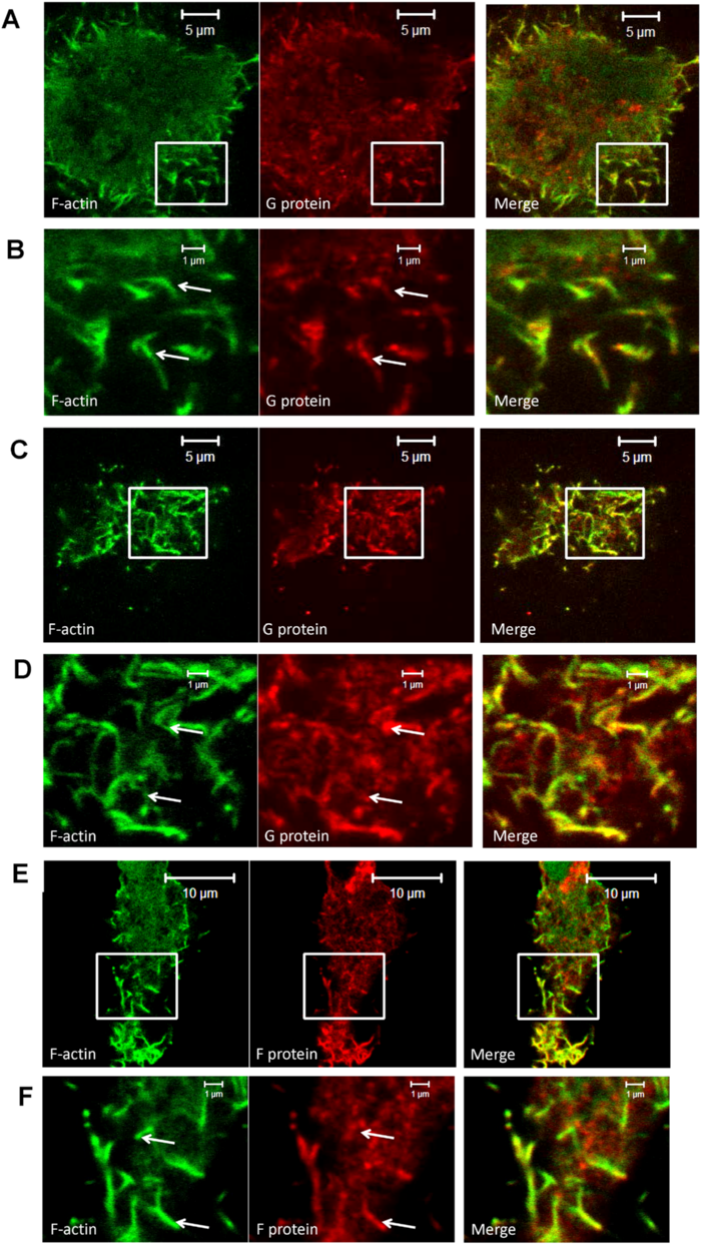
**Co-localisation of recombinant expressed G and F proteins with phalloidin-FITC in LLC-MK2 cells. (A to D)** LLC-MK2 cells were transfected with pCAGGS/G-FLAG and at 14 hrs post-transfection the cells were stained using anti-FLAG and phalloidin-FITC, and examined by confocal microscopy at a focal plane that allows **(A and B)** the cell periphery and **(C and D)** the cell top to be visualised. **(B)** is an enlarged region from plate **(A)** (highlighted by white box) and **(D)** is an enlarged region from plate **(C)** (highlighted by white box). **(E and F)** LLC-MK2 cells were co-transfected with pCAGGS/G-FLAG and pCAGGS/F-cmyc and at 14 hrs post-transfection the cells were stained using anti-cmyc and phalloidin-FITC. **(F)** is an enlarged region from plate **(E)** (highlighted by white box). In each plate the filamentous staining pattern is highlighted (white arrows).

**Figure 6:**
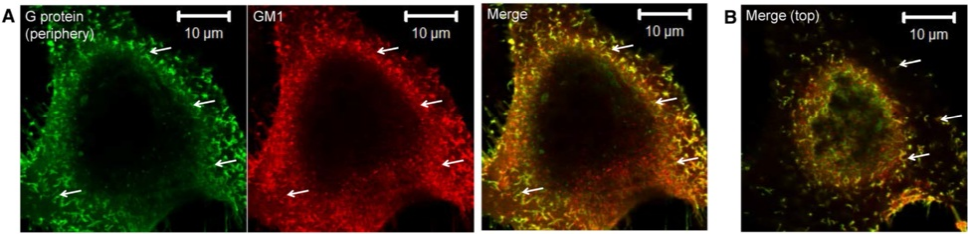
**Co-localisation of recombinant expressed G protein and GM1 in LLC-MK2 cells.** LLC-MK2 cells were transfected with pCAGGS/G-FLAG and at 14 hrs-post transfection the cells were stained using anti-FLAG and CTX-B-AF594 and examined by confocal microscopy at a focal plane that allows **(A)** the cell periphery and **(B)** the cell top to be viewed. In each plate the filamentous staining pattern is highlighted (white arrows).

We therefore used this recombinant expression system to examine the relative distribution of the G protein, GM1 and F-actin in cells expressing recombinant G protein. Cells were transfected with pCAGGS/G-Flag and at 14 hrs post-transfection the cells were co-stained with anti-Flag (to detect the G protein) and phalloidin-FITC (Figure 5A-D). Similarly, cells were co-transfected with pCAGGS/G-Flag and pCAGGS/F-cmyc and stained with anti-cmyc (to detect the F protein) and phalloidin-FITC (Figure 5E and F). In both cases a prominent filamentous G and F protein staining pattern was observed as described previously [23], and in both cases we noted co-staining of the G and F protein filaments with phalloidin-FITC. Similarly cells were transfected with pCAGGS/G-Flag and at 14 hrs post-transfection the cells were co-stained with anti-Flag and CTX-B-AF594 (Figure 6A and B). A prominent filamentous G protein staining pattern was observed, and we noted extensive co-staining of the G protein filaments with CTX-B-AF594.

**Figure 7:**
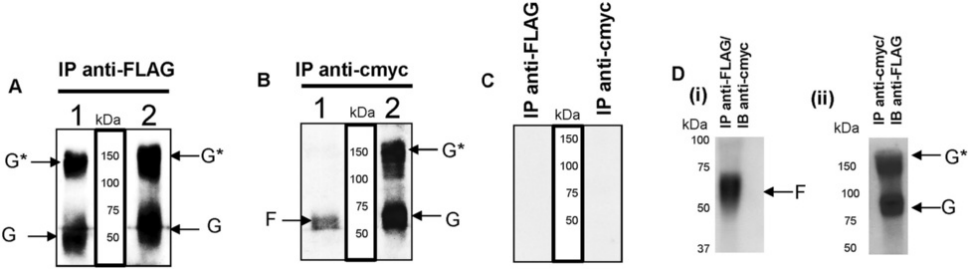
**Biochemical evidence for an interaction between the F and G proteins on co-transfected cells. (A)** Cells were either transfected with either (1) pCAGGS/G-FLAG or (2) pCAGGS/G-FLAG and pCAGGS/F-cmyc, and then surface-biotinylated and immunoprecipitated (IP) with anti-FLAG or **(B)** cells were either transfected with either (1) pCAGGS/F-cmyc or (2) pCAGGS/G-FLAG and pCAGGS/F-cmyc, and then surface biotinylated and immunoprecipitated (IP) with anti-cmyc. **(C)** Cells were transfected with pCAGGS, and then surface-biotinylated, and immunoprecipitated (IP) with either anti-FLAG or anti-cymc. Biotinylated proteins species corresponding to the G and F proteins are indicated. **(D) (i)** pCAGGS/G-FLAG and pCAGGS/F-cmyc, co-transfected cells were immunoprecipitated (IP) with anti-FLAG and immunoblotted (IB) with anti-cmyc or **(ii)** cells were immunoprecipitated (IP) with anti-cmyc and immunoblotted with anti-FLAG. Proteins species corresponding to the G protein (G and G*) and F protein (F) are indicated.

We confirmed the interaction between the F and G proteins by co-immunoprecipitation assay (Figure 7). The cells were transfected singly with pCAGGS/G-Flag or pCAGGS/F-cmyc, or alternatively co-transfected with pCAGGS/G-Flag and pCAGGS/F-cmyc, and after 14 hr post-tranfection the cells were surface-biotinylated (Figure 7A and B). Cells expressing only the F protein or G protein immunoprecipitated with either anti-FLAG or anti-cmyc respectively showed biotinylated protein species corresponding in size to the G and F proteins respectively. In lysates prepared using cells co-expressing the pCAGGS/G-Flag and pCAGGS/F-cmyc and immunoprecipitated with anti-FLAG a protein species corresponding in size to the G protein was observed. In contrast immunoprecipitation with anti-cmyc also showed protein species corresponding in size to the G protein. This was consistent with an interaction between the F and G proteins on the surface of co-transfected cells. As observed previously [23], we also noted that the G protein expressed in co-transfected cells exhibited a slightly larger apparent mass compared to that in cells expressing only the G protein. We originally interpreted this observation as evidence that co-expression of the F protein influenced the processing of the G protein, providing additional evidence of an interaction. We failed to detect the presence of biotinylated proteins in cells transfected with the parent expression vector and immunoprecipitated with either anti-FLAG or anti-cmyc (Figure 7C). Co-precipitation of the G protein following immunoprecipitation with anti-cmyc and F protein following immunoprecipitation with anti-FLAG was confirmed by immunoblotting with anti-FLAG and anti-cmyc respectively (Figure 7D). These observations suggested the formation of a single protein complex between the F and G proteins in the co-transfected cells, confirming our earlier observations [23].

**Figure 8:**
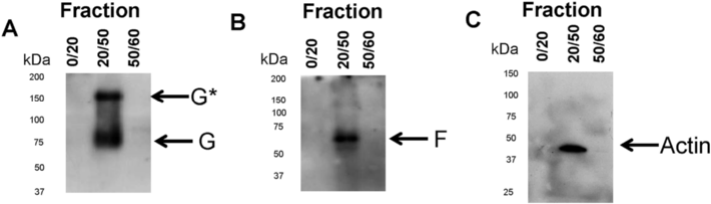
**The VLPs prepared in pCAGGS/G-FLAG and pCAGGS/F-cmyc co-transfected cells contained actin.** Cells were co-transfected with pCAGGS/G-FLAG and pCAGGS/F-cmyc, and VLPs prepared by discontinuous centrifugation as described in methods. The 0–20, 20–50 and 50-60% sucrose interface fractions were examined by immunoblotting using **(A)** anti-FLAG, **(B)** anti-cmyc and **(C)** anti-actin. Protein species corresponding in size to the recombinant G-FLAG and F-cmyc proteins and actin are indicated. G indicates monomeric G protein and G* is a larger G protein species.

The imaging analysis suggested that the protein complex formed by the F and G proteins is located within F-actin stabilised cellular structures on the surface of co-transfected cells. We have recently demonstrated that the HMPV VLPs could be isolated by ultracentrifugation using a 20%(w/v)sucrose, 50%(w/v)sucrose, and 60%(w/v)sucrose discontinuous gradient (23). In this earlier study we had used this discontinuous sucrose gradient centrifugation methodology as an assay with which to monitor VLP formation and identify virus determinants of VLP formation (23). Cells were co-transfected with pCAGGS/G-Flag and pCAGGS/F-cmyc and after 24 hr post-transfection the cells were harvested and VLPs prepared as described in methods. The gradient was fractionated and the interfaces between the different sucrose concentrations examined by immunoblotting using anti-cmyc and anti-FLAG to detect the presence of the F-myc and G-FLAG respectively. Similarly, we examined the different fractions by immunobloting using anti-actin to determine the presence of actin. Immunoblotting with anti-FLAG (Figure 8A), and anti-cmyc (Figure 8B) revealed the presence of the HMPV G and F proteins in the 20/50(w/v) sucrose interface as we have demonstrated previously (23). In addition, immunoblotting of these fractions with anti-actin (Figure 8C) showed the presence of actin in the same fraction as the HMPV glycoproteins i.e. at the 20/50(w/v) sucrose interface. This provides additional biochemical evidence that the VLPs are formed within the F-actin structures identified in the imaging analysis of the co-transfected cells.

These data indicated that the G protein is able to transport to regions of the cell membrane that are enriched in F-actin and GM1 independently of other virus proteins. As suggested in our previous study, the filamentous staining pattern exhibited by the HMPV G protein indicates the presence of VLPs rather than the filamentous particles that are produced in HMPV-infected cells. We propose that the VLPs allow the identification of the sites of HMPV particle assembly in LLC-MK2 cells. This further suggested that the HMPV G protein contains the necessary targeting signals that can traffic the protein into the sites of HMPV particle assembly, locations where lipid-raft microdomains and F-actin are enriched.

## Conclusion

Our observations indicate that the HMPV virus matures with a filamentous morphology in a manner similar to that described for RSV. Several studies have suggested a role for F-actin in RSV particle formation [17,30]. F-actin is also present at the site of HMPV assembly, and the correlation between HMPV assembly and the presence of F-actin suggests that F-actin may also play a role in the formation of HMPV filaments.

Lipid-raft membranes exist as a liquid crystalline phase, leading to formation of stabilized lipid structures within the bulk lipid membrane [31]. The incorporation of cholesterol-dependent raft structures into the influenza virus envelope correlates with a reduction in the ‘fluidity’ of the viral envelope [32]. The presence of raft-lipids in the envelope of HMPV filaments suggested the presence of similar highly ordered lipid microdomains (lipid-rafts) within the viral envelope. This further suggests that these lipids may impart important structural and functional properties to the HMPV envelope.

The low virus yields and slow replication rates of HMPV in tissue culture are major impediments to understanding HMPV morphogenesis using standard biochemical methodology. However, in this study we have used imaging to examine the distribution of virus structural proteins and specific cellular markers during HMPV maturation in individual virus-infected cells. This experimental approach has provided the first direct evidence to suggest a role for lipid-raft membranes and F-actin in the process of HMPV morphogenesis. Interestingly, the similarity between HMPV and the closely related RSV suggests that the formation of virus filaments may be a common feature of virus morphogenesis for other viruses within the family *Pneumovirinae*. Our analysis also highlights the utility of *in situ* imaging using specific virus and cellular markers to examine the morphogenesis of viruses that are not easy to propagate in standard tissue culture. This alternative strategy should become increasingly accessible as the number of commercially available immunological reagents and cellular probes increases.

## Materials and methods

### Virus and cells

The HMPV A2 strain NCL03-4/174 was described previously [13]. The HMPV was used to infect the LLC-MK2 cell line in DMEM (BSA, 0.5 μg/ml TPKC-trypsin) at 37°C. The LLC-MK2 cell line was maintained in DMEM with 10% FCS at 37°C.

#### Antibodies used

The antibodies Mab58 (anti-F) and AT1 (anti-G) have been described previously [16]. The tagged HMPV proteins were detected with rabbit anti-FLAG antibodies (Sigma-Aldrich, USA), mouse anti-cmyc antibodies (Cell Signaling Technology, USA). The anti-M was prepared using recombinant expressed M protein. Briefly, the HMPV M gene was cloned into pRSETB and expressed in 1 mM IPTG-induced *E.coli* as his-tagged proteins. After 6 hr induction the bacterial cells were lysed and the recombinant HMPV proteins recovered from the insoluble lysed bacterial pellet using 6 M GuHcl (1 mM EDTA, 100 mM NaCl, 10 mM Tris-Cl pH 8). The proteins were bound to nickel agarose using the manufacture’s protocol (Qiagen), and the recombinant proteins were eluted using 8 M urea in 250 mM imidazole, 1 mM EDTA, 100 mM NaCl, 10 mM Tris-Cl pH 8, and diluted into refolding buffer (1 mM EDTA, 2 mM PMSF, 100 mM NaCl, 10 mM Tris-Cl pH 8). The resulting eluted protein solution was concentrated and used to immunise BALB/c mice and monoclonal antibodies prepared using standard procedures.

#### Expression of G-FLAG and F-cmyc proteins in transfected LLC-MK2 cells

The G and F gene were amplified from the HMPV A2 positive-nasopharyngeal washings (SIN06-NTU271) and inserted into the vector pCAGGS [33] to generate pCAGGS/G-FLAG and pCAGGS/F-cmyc respectively as described previously [23]. Cells were transfected using Lipofectamine 2000 reagent (Invitrogen, USA) following the manufacturer’s instructions.

#### Western blotting

The proteins were separated by SDS–PAGE, transferred onto PVDF membranes (Immobilon-P, Milipore, USA) as described previously [29]. Protein bands were visualised using the ECL system (GE Healthcare, USA). Molecular masses were estimated using Kaleidoscope markers (Biorad, USA).

#### Immunoprecipitation

Cell extracts were prepared at 4°C using RIP buffer (1%NP-40, 0.1%SDS, 150 mM NaCl, 1 mM EDTA, 2 mM PMSF, 2 mM lysine, 20 mM Tris–HCl, pH7.5) and clarified by centrifugation (13,000 g, 10 min 4°C) and immunoprecipitated as described previously [18] using appropriate antibodies. The immunoprecipitation assays were separated using SDS-PAGE.

#### Surface labelling

Cells were surface-labelled using EZ-Link Sulfo-NHS-LC-LC-Biotin (Pierce Biotechnology, USA) as described previously [18]. Briefly, cell monolayers were incubated in 0.5 mg/ml solution of EZ-Link Sulfo-NHS-LC-LC-Biotin (Pierce Biotechnology, USA) in PBS pH 8 for 1 hr at room temperature. The lysates were immunoprecipitated using the appropriate antibody.

#### Immunofluorescence and confocal microscopy

Cells were fixed with 4% PFA or methanol:acetone (1:1) for 15 min at 4°C and the cells were labelled using the appropriate primary and secondary antibody (conjugated to either FITC or Alexa Fluor 555). Staining with phalloidin-FITC (Sigma) and cholera toxin-B subunit conjugated to AF488 or AF594 (invitrogen) was also performed as described previously [24]. The cells were visualized using either a Nikon eclipse 80i fluorescence microscope (Nikon ECLIPSE TE2000-U) or a Zeiss Axioplan 2 confocal microscope using appropriate machine settings.

#### Isolation of VLPs

This was performed as described previously (23). Briefly, cell suspension was subjected to freeze-thaw, the cell suspension was clarified (2,500 g for 10 min) and loaded onto a sucrose cushion (10%w/v sucrose in TEN buffer), and centrifuged at 200,000 g for 1 hr at 4°C (Hitachi CP90WX ultracentrifuge). The resulting pellet was resuspended in 200 μl of TEN buffer and loaded onto a discontinuous sucrose gradient (20%, 50% and 60% sucrose (w/v) in TEN buffer). The material was harvested from each sucrose interface and used for further analysis.

## Additional files

Additional file 1: Figure S1. Time course study of LLC-MK2 cells infected with human metapneumovirus (HMPV). **(A)** LLC-MK2 cells were infected with HMPV and at 3 days (3 dpi), 7 days (7 dpi) and 10 days post-infection (10 dpi) the cells were fixed and stained with anti-M. Virus antigens were detected by immunofluorescence microscopy (anti-M). Infected cells clusters are highlighted (white arrows) (x20 objective). **(B)** LLC-MK2 cells were infected with HMPV and at 7 dpi the cell monolayer was stained using anti-G and Evans Blue (EB) (to visualise the cell monolayer). HMPV filaments are indicated (white arrows). Additional file 2: Figure S2. Analysis of the surface topology of mock-infected and HMPV-infected LLC-MK2 cells using scanning electron microscopy (SEM). At 7 days post-infection mock-infected and HMPV-infected LLC-MK2 cells (grown on 10 mm glass coverslips) were processed for SEM as described previously [19,21]. Briefly, the cells were incubated in the primary fixative (3%(v/v) glutaraldehyde in PBS, washed extensively in PBS and then incubated in the secondary fixative (1% osmium tetroxide). The cells were dehydrated using a 0-100% (v/v) ethanol gradient and critical point-dried (Polaron CPD) using ethanol. The cells were mounted on aluminium stubs and gold-coated. The processed cells were visualized with a Quanta FEG 200 (FEI) scanning electron microscope using appropriate machine settings (magnification x20,000). The presence of microvilli (mv), virus filaments (VF) and spherical bodies (S) are highlighted.

## Abbreviations

HMPV: Human metapneumovirus
RSV: Respiratory syncytial virus
VLPs: Virus-like particles
CTX-B-AF488: Cholera toxin B-subunit conjugated to AF488
F protein: Fusion protein
G protein: Attachment protein
SEM: Scanning electron microscopy
FEG: Field emission gun
G-FLAG: G protein with a c-terminal FLAG tag
F-cmyc: F protein with a c-terminal cmyc tag
