# Supplementary figures and images for: Imaging analysis of human metapneumovirus-infected cells provides evidence for the involvement of F-actin and the raft-lipid microdomains in virus morphogenesis
Source: Virol J. 2014 Nov 19;11:198. doi: 10.1186/s12985-014-0198-8 (PMC4243936; doi:10.1186/s12985-014-0198-8)

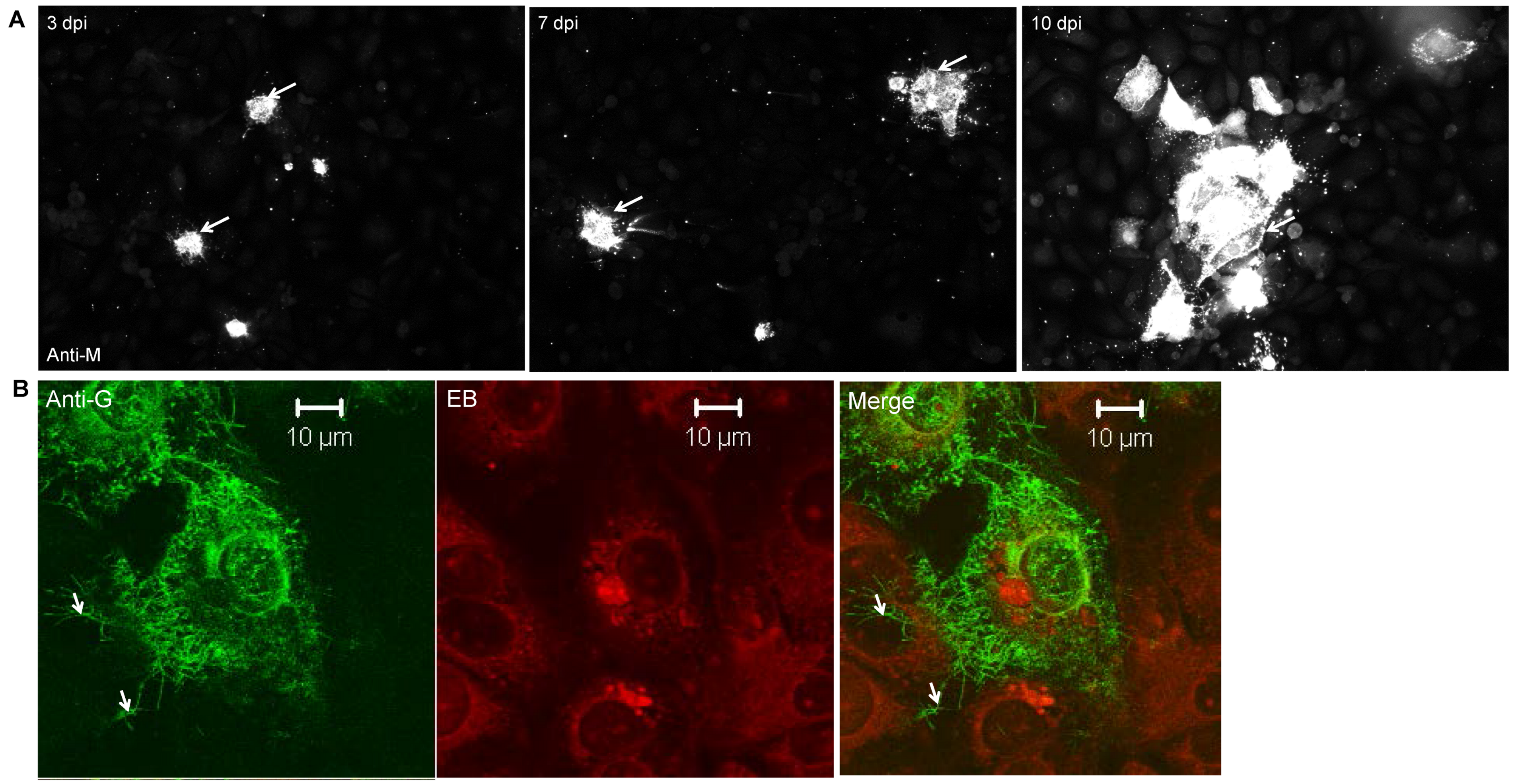

Supplement: Additional file 1: Figure S1. — Time course study of LLC-MK2 cells infected with human metapneumovirus (HMPV). (A) LLC-MK2 cells were infected with HMPV and at 3 days (3 dpi), 7 days (7 dpi) and 10 days post-infection (10 dpi) the cells were fixed and stained with anti-M. Virus antigens were detected by immunofluorescence microscopy (anti-M). Infected cells clusters are highlighted (white arrows) (x20 objective). (B) LLC-MK2 cells were infected with HMPV and at 7 dpi the cell monolayer was stained using anti-G and Evans Blue (EB) (to visualise the cell monolayer). HMPV filaments are indicated (white arrows). [file 12985_2014_198_MOESM1_ESM.tiff]

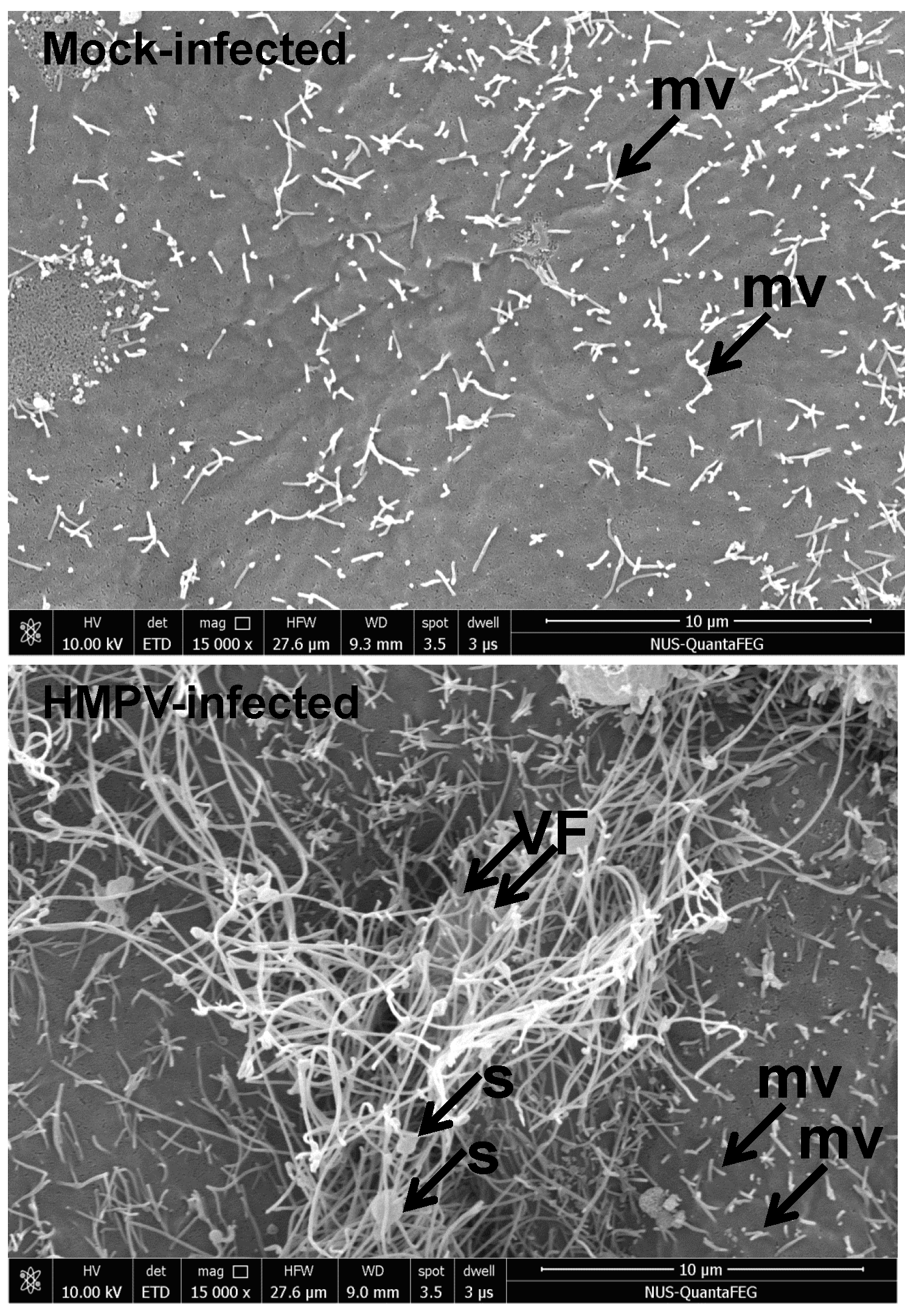

Supplement: Additional file 2: Figure S2. — Analysis of the surface topology of mock-infected and HMPV-infected LLC-MK2 cells using scanning electron microscopy (SEM). At 7 days post-infection mock-infected and HMPV-infected LLC-MK2 cells (grown on 10 mm glass coverslips) were processed for SEM as described previously [19,21]. Briefly, the cells were incubated in the primary fixative (3%(v/v) glutaraldehyde in PBS, washed extensively in PBS and then incubated in the secondary fixative (1% osmium tetroxide). The cells were dehydrated using a 0-100% (v/v) ethanol gradient and critical point-dried (Polaron CPD) using ethanol. The cells were mounted on aluminium stubs and gold-coated. The processed cells were visualized with a Quanta FEG 200 (FEI) scanning electron microscope using appropriate machine settings (magnification x20,000). The presence of microvilli (mv), virus filaments (VF) and spherical bodies (S) are highlighted. [file 12985_2014_198_MOESM2_ESM.tiff]
